# Molecular subtypes of ischemic heart disease based on circadian rhythm

**DOI:** 10.1038/s41598-024-65236-5

**Published:** 2024-06-19

**Authors:** Zhaokai Zhou, Ge Zhang, Zhan Wang, Yudi Xu, Hongzhuo Qin, Haonan Zhang, Pengpeng Zhang, Zhengrui Li, Shuai Xu, Xin Tan, Yiyao Zeng, Fengyi Yu, Shanshan Zhu, Le Chang, Youyang Zheng, Xinwei Han

**Affiliations:** 1 Department of Interventional Radiology, The First Affiliated Hospital of Zhengzhou University, Zhengzhou, 450052 Henan China; 2 Department of Urology, The First Affiliated Hospital of Zhengzhou University, Zhengzhou, 450052 Henan China; 3 Department of Cardiology, The First Affiliated Hospital of Zhengzhou University, Zhengzhou, 450052 Henan China; 4 Department of Neurology, The First Affiliated Hospital of Zhengzhou University, Zhengzhou, 450052 Henan China; 5 Department of Thyroid Surgery, The First Affiliated Hospital of Zhengzhou University, Zhengzhou, China; 6 Department of Lung Cancer, Tianjin Lung Cancer Center, National Clinical Research Center for Cancer, Key Laboratory of Cancer Prevention and Therapy, Tianjin’s Clinical Research Center for Cancer, Tianjin Medical University Cancer Institute and Hospital, Tianjin, China; 7 Department of Oral and Maxillofacial-Head and Neck Oncology, Shanghai Ninth People’s Hospital, Shanghai Jiao Tong University School of Medicine, Shanghai, China; 8 Department of Cardiology, The Fourth Affiliated Hospital of Soochow University, Suzhou Dushu Lake Hospital, Medical Center of Soochow University, Suzhou, 215000 China; 9 Institute for Hypertension, Soochow University, Suzhou, 215000 China; 10 Department of Gastroenterology, The First Affiliated Hospital of Zhengzhou University, No. 1 Jianshe East Road, Zhengzhou, China; 11 School of Medicine, Zhengzhou University, Zhengzhou, China; 12 Department of Cardiology, Fuwai Hospital, National Centre for Cardiovascular Diseases, National Clinical Research Centre for Cardiovascular Diseases, Chinese Academy of Medical Sciences and Peking Union Medical College, Beijing, China; 13 Interventional Institute of Zhengzhou University, Zhengzhou, 450052 Henan China; 14 Interventional Treatment and Clinical Research Center of Henan Province, Zhengzhou, 450052 Henan China

**Keywords:** Bioinformatics, Circadian rhythm, Ischemic heart disease, Transcriptome, Cardiology, Computational biology and bioinformatics, Gene ontology

## Abstract

Coronary atherosclerotic heart disease (CAD) is among the most prevalent chronic diseases globally. Circadian rhythm disruption (CRD) is closely associated with the progression of various diseases. However, the precise role of CRD in the development of CAD remains to be elucidated. The Circadian rhythm disruption score (CRDscore) was employed to quantitatively assess the level of CRD in CAD samples. Our investigation revealed a significant association between high CRDscore and adverse prognosis in CAD patients, along with a substantial correlation with CAD progression. Remarkably distinct CRDscore distributions were also identified among various subtypes. In summary, we have pioneered the revelation of the relationship between CRD and CAD at the single-cell level and established reliable markers for the development, treatment, and prognosis of CAD. A deeper understanding of these mechanisms may offer new possibilities for incorporating "the therapy of coronary heart disease based circadian rhythm" into personalized medical treatment regimens.

## Introduction

Coronary atherosclerotic heart disease (CAD) is an atherosclerotic disease that is inflammatory, manifested by stable angina, unstable angina, myocardial infarction (MI), or sudden cardiac death, which is one of the major cardiovascular diseases affecting the global human population^1^. This disease has been proven to be the major cause of death in both the developed and developing countries^2^. Lifestyle, environmental, and genetic factors pose risk factors for the development of cardiovascular disease like coronary artery disease^3,4^. The prevalence of risk factors among healthy individuals elucidates the probable occurrence of CAD. It has been demonstrated that the progression and prognosis of CAD are closely related to the formation of vulnerable atherosclerotic plaques^5^. Anatomicopathological studies established characteristics of vulnerable atherosclerotic plaques, including a thin, fibrous cap and a large lipid core populated by numerous inflammatory cells and relatively lacking in smooth muscle cells^6^.

The circadian rhythm timing system governs most physiological processes in mammals, and disrupted circadian clocks are emerging as novel indicators influencing disease incidence and progression^7^. Evidence of circadian rhythm disruption (CRD) has been found in various diseases, mostly focused on cancer, including colorectal cancer, lung cancer, melanomas, and breast cancer^8–11^. In addition, studies have shown that CRD is closely related to the progression of CAD^12,13^. Variations in circadian rhythms are evident in the incidence of cardiovascular disease, and the risk of cardiovascular events increases when rhythms are disrupted. Recent studies have shown that circadian rhythms exist in myocardial tissue and are involved in metabolism and contractile function^14,15^. Epidemiologic studies also demonstrated the existence of circadian patterns in the incidence of cardiovascular disease. For example, the onset of non-Q-wave angina, unstable angina, MI, and sudden cardiac death all show marked elevations in the occurrence between the hours of 6:00 am and 12:00 pm, compared with any other time of day^12^. Additionally, variables such as blood pressure (BP), heart rate (HR), peripheral resistance, and the release/activity of vasodilating hormones all display pronounced circadian fluctuations^16^. A better understanding of the function of CRD genes in the heart and response to injury may contribute to the development of innovative therapies for cardiovascular disease^17,18^. Therefore, it is important to account for circadian rhythms as a key research parameter of cardiac physiology/pathology.

Currently, the development of computational biology and highly sophisticated technologies like high-throughput sequencing technologies accelerate our understanding of CAD. Nevertheless, an explanation of CAD behavior based on circadian rhythms at the omics and systems biology levels has not yet emerged. Therefore, we employed the CRDscore algorithm to directly infer single-cell-level CRD states from the transcriptome profiles of CRD-related genes. Our results indicated that vulnerable atherosclerotic plaques exhibited consistently higher CRDscore than stable atherosclerotic plaques. Importantly, CRDscore was associated with adverse prognosis in CAD patients. Furthermore, we used expression profiles of CRD-related genes from vulnerable atherosclerotic plaques to build accurate and robust models to classify patients and predict patient outcomes to implement personalized treatment measures. In summary, we have pioneered the revelation of CRD genes closely associated with the process of CAD at the single-cell level and established reliable markers for the development, treatment, and prognosis of CAD.

## Materials and methods

### Data processing

We systematically retrieved publicly available and clinically annotated single-cell RNA sequencing (scRNA-seq) datasets related to CAD. The dataset selection process followed these criteria in PubMed: (((Coronary artery disease[Title]) OR (Coronary heart disease[Title])) OR (CAD[Title/Abstract])) OR (CAD[Title/Abstract])) AND (((single‐cell transcript*[Title/Abstract]) OR (single‐cell RNA[Title/Abstract])) OR (scRNA‐seq[Title/Abstract])) OR (cell type*[Title/Abstract]).

We obtained and analyzed the GSE184073 datasets (Illumina NovaSeq 6000, high throughput sequencing) from the Gene Expression Omnibus (GEO, www.ncbi.nlm.nih.gov/geo/) database, including one sample with stable angina pectoris and one sample with acute coronary syndrome^19^. Using the Seurat R package, we generated Seurat objects containing scRNA-seq gene expression matrices for specific cell types. Batch correction and sample integration were executed using the IntegrateData function of the Seurat R package^20^. The top 2000 variable genes were identified through the FindVariableFeatures function from the Seurat package. Cells expressing between 200 and 4000 genes and with less than 15% mitochondrial genes were chosen. The remaining scRNA-seq data underwent normalization and scaling for subsequent analysis, achieved via the NormalizeData and ScaleData functions from the Seurat R package. The number of principal components (PCs) was determined using the RunPCA function, followed by dimensionality reduction analysis conducted using Uniform Manifold Approximation and Projection (UMAP) or t-distributed Stochastic Neighbor Embedding (t-SNE)^19,21,22^. Individual cell clusters were labeled to identify cell populations by checking the gene expression of well-defined cell markers, with the CellMarker database and singleR as a reference (http://117.50.127.228/CellMarker/). Moreover, the *Cell Cycle Scoring* function in Seurat was used for cell cycle discrimination analysis and quantification, based on previously defined cell cycle-related genes^23^.

To assess the robustness and clinical performance of transcriptome-based CRD scores, we acquired 4 GEO datasets comprising GSE10045 (Illumina Mouse Ref-6 V1 Microarray data, n = 130), GSE70049 (Zebrafish Gene 1.0 ST Microarray, n = 6), GSE43073 (Illumina MouseRef-8 v2.0 expression beadchip, Microarray data, n = 64), and GSE35026 (Affymetrix Mouse Gene 1.0 ST Microarray, n = 24). Additionally, 436 samples from GSE59867 (Affymetrix Human Gene 1.0 ST Microarray) constituted the discovery/training cohort in consensus typing. The validation/testing cohorts consisted of GSE20680 (Agilent-014850 Whole Human Genome Microarray, n = 195), GSE20681 (Agilent-014850 Whole Human Genome Microarray, n = 198), GSE43292 (Affymetrix Human Gene 1.0 ST Array, n = 64), and GSE62646 (Affymetrix Human Gene 1.0 ST Array, n = 98). For microarray data, we conducted robust multi-array averaging (RMA) normalization to standardize the microarray signals. RNA-seq data, in the form of counts or FPKM values, was imported into R for further analysis, beginning with transcript per kilobase million (TPM) values that underwent log2 transformation^24^.

### Single cell co-expression network analysis

The Single Cell Co-Expression Network Analysis (SCCNA) was harnessed to analyze single-cell sequencing data^25^. The initial step involved optimizing the data preprocessing by creating a "Shrinkage object" using carefully selected parameters. Subsequently, we constructed a co-expression network characterized by a soft threshold. Leveraging the concept of feature genes, we conducted a meticulous evaluation of gene connectivity, namely module eigengenes' interconnectivity (KME) to identify hub genes.

### Calculation of CRDscore based on circadian-related genes

In this study, we implemented the CRDscore algorithm to obtain CRD-related scores, utilizing the expression profiles of coronary atherosclerotic heart disease circadian rhythm genes (CADCR genes)^26,27^. Our methodology encompassed three principal steps:

Firstly, we computed the TPM values for scRNA-seq data and normalized values on bulk microarray for CAD. Secondly, implementing a random sampling strategy, we selected genes from each bin with randomly assigned labels. This approach aimed to mitigate sampling bias. Lastly, we constructed a central gene expression matrix, representing data devoid of excessive signal shifts. To quantify the abundance of cell rhythmic genes (CRGs), we assigned a random score, denoted as Srandom, to each sample or cell. This score represents the average of 1000 random features obtained through the aforementioned sampling process. In addition, the average score among CRGs was used as the CRG score, namely SCADCRgenes. The CRDscore is computed as follows:

$${\text{CRDscore}} = {\text{Srandom}} - {\text{SCADCRgenes}}$$

For single-cell data, the quartile-based 75th percentile cutoff was used as the threshold for CRD levels, while the median was used as the threshold for CRD levels in the bulk transcriptome data.

### Construction and validation of the CRD-related subtypes of CAD

To investigate CRD patterns in CAD, unsupervised clustering based on gene sets of the M1 module was conducted for GSE59867 datasets via “ConsensusClusterPlus” R package^28^. The method was built as a K-means algorithm based on Euclidean distance with 1000 iterations to ensure the stability of the classification, with the number of clusters set from 2 to 6. The repeatability and robustness of the proposed subtypes were further quantitatively assessed in a validation cohort using the nearest template prediction (NTP) algorithm. NTP has been widely used for signature gene expression-based typing and confidence assessment^29,30^. Additionally, we downloaded 4 independent datasets from GEO databases to verify the stability and robustness of typing, including GSE20680, GSE20681, GSE43292, and GSE62646.

### Function enrichment analysis

The enrichment analysis was conducted to decode the underlying biological molecular mechanisms of different CAD-related subtypes via the "clusterProfiler” R package^31^. Our study retrospectively extracted 7844 annotated gene sets from the Gene Ontology (GO, n = 7658) and Kyoto Encyclopedia of Genes and Genomes (KEGG, n = 186)^32–34^. Gene set variation analysis (GSVA) was a type of gene set enrichment method measuring the enrichment degree of different pathways among different samples via GSVA R package^35^. To identify the correlated pathways underlying four subtypes, we employed GSVA of the “Hallmark” genesets.

### Immune microenvironment analysis

xCell is a web tool (http://xCell.ucsf.edu/) that performs cell type enrichment analysis from gene expression data for 64 immune and stroma cell types. xCell is a gene signatures-based method learned from thousands of pure cell types from various sources. We applied xCell to observe differences in immune and stromal cell infiltration among the subtypes^36,37^.

### Quantitative and statistical analysis

All statistical tests conducted in this study were two-sided. Statistical significance was defined as a P-value < 0.05 and a False Discovery Rate (FDR) < 0.05. Descriptive statistics, including mean and standard deviation (SD), were employed for continuous variables exhibiting a normal distributio^38^. Wilcoxon rank-sum and t-tests were employed for comparing continuous variables, while chi-square tests or Fisher's exact tests were used for categorical variables. Data processing, statistical analyses, and visualization were carried out using R 4.2.0 software.

## Results

### Construction of WGCNA network in scRNA-seq dataset

**Figure 1:**
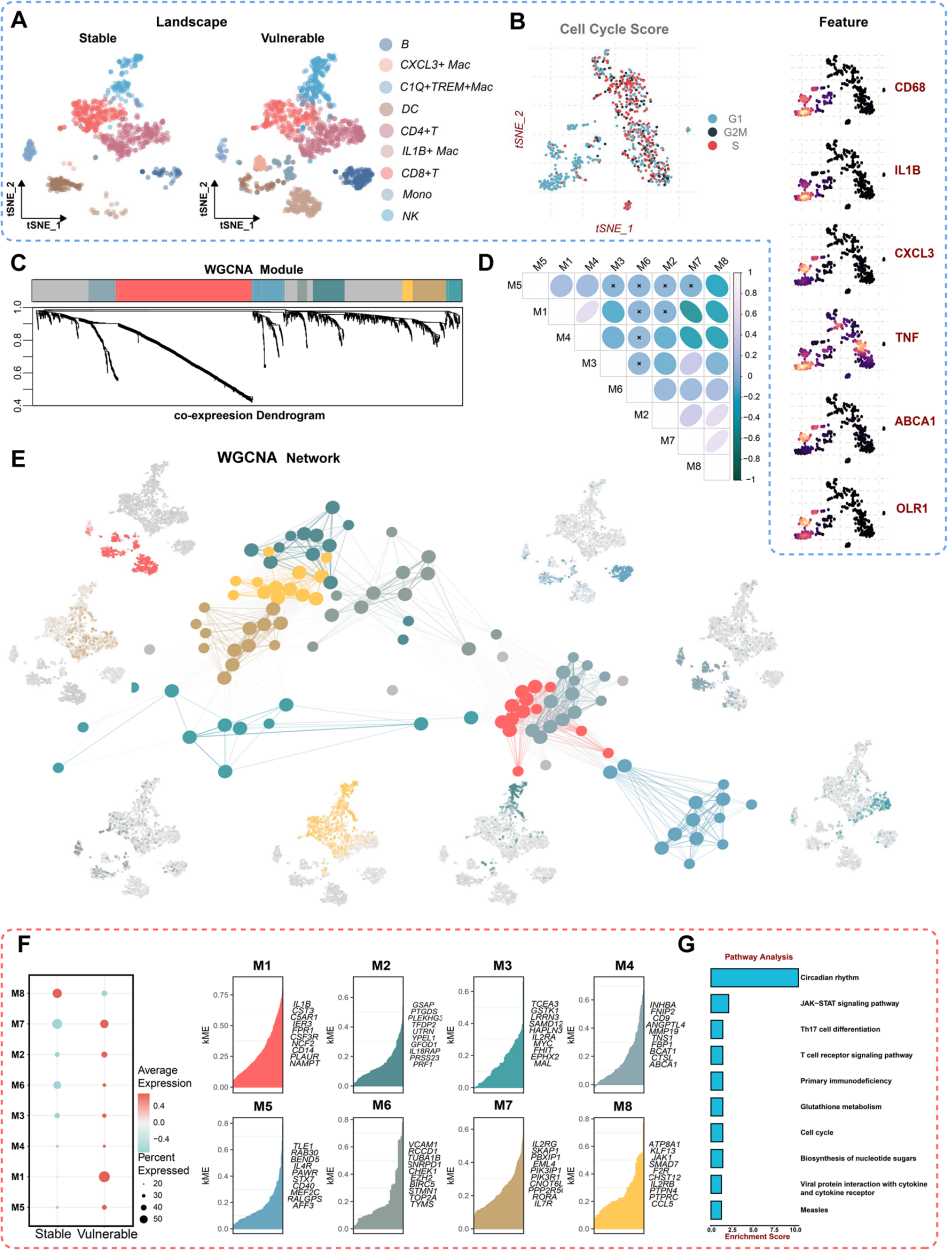
Preliminary Screening of Vulnerable plaques-related Genes Using SCCNA. (**A**) tSNE visualization of cell type annotations of stable plaques and vulnerable atherosclerotic plaques. (**B**) tSNE visualization of cell cycle score and marker genes of vulnerable atherosclerotic plaques. (**C**) Co-expression dendrogram of WGCNA module. (**D**) Correlation diagram for 8 modules. (**E**) UMAP visualization of all genes in the co-expression network. (**F**) Scatter plot displaying the average expression of module-specific hub genes in stable plaques and vulnerable atherosclerotic plaques (Left). Heatmap of KME value of hub genes for each module (Right). (**G**) Function enrichment analysis of M1 and M7 modules.

To delve into the underlying mechanisms of CRD during the development of CAD, we meticulously curated and extracted coronary atherosclerosis cells from the scRNA-seq dataset (GSE184073). The amalgamation of data yielded a total of 2,237 cells from two samples (one sample with stable angina pectoris and one sample with acute coronary syndrome) following stringent quality control and filtering. Next, we performed the following procedure on the scRNA-seq data of the two samples respectively. Following gene expression normalization, we embarked on Principal Component Analysis (PCA) and subsequently clustered cells within the PCA space using t-distributed stochastic neighbor embedding (tSNE). These results identified 9 discrete cell clusters exhibiting differential gene expression (DEG). These cell clusters were categorized into known cell lineages based on distinctive gene markers, encompassing macrophage (CXCL3^+^ Mac cells, C1Q^+^TREM^+^ Mac cells, IL1B^+^ Mac cells), CD4^+^ T cells, CD8^+^ T cells, B cells, natural killer (NK) cells, dendritic cells (DCs), and monocyte (Fig. 1A). By t-SNE visualization of cell cycle score for 2,237 cells, we found that multiple immunoinflammatory-associated and lipid metabolism-related genes were enriched in the G1 phase, such as CD68, interleukin-1 beta (IL1B), chemokine (C-X-C motif) ligand 3 (CXCL3), tumor necrosis factor (TNF), ABCA1, and oxidized low-density lipoprotein receptor 1 (OLR1) suggesting that inflammatory response played an important role in early cell proliferation in CAD (Fig. 1B).

Subsequently, we conducted the hbWGCNA within the integrated scRNA-seq dataset. An optimal soft threshold of 12 was employed to craft a co-expression network grounded in single-cell data. The eight non-grey modules were identified through co-expression network analysis along with their correlations (Fig. 1C,D). Module 1 has the strongest correlation with module 7. UMAP dimensionality reduction was harnessed to simultaneously visualize all genes within the co-expression network (Fig. 1E). Notably, Fig. 1F elucidated the differences in co-expression modules between two cell groups (stable and vulnerable plaques) in the scRNA-seq dataset. Module 1 and Module 7 exhibited significant overlap with vulnerable plaque cells (Fig. 1F). Functional enrichment analysis of genes from the M1 and M7 modules underscored their involvement in circadian rhythms (Fig. 1G).

### Effectiveness and robustness of CRDscore

**Figure 2:**
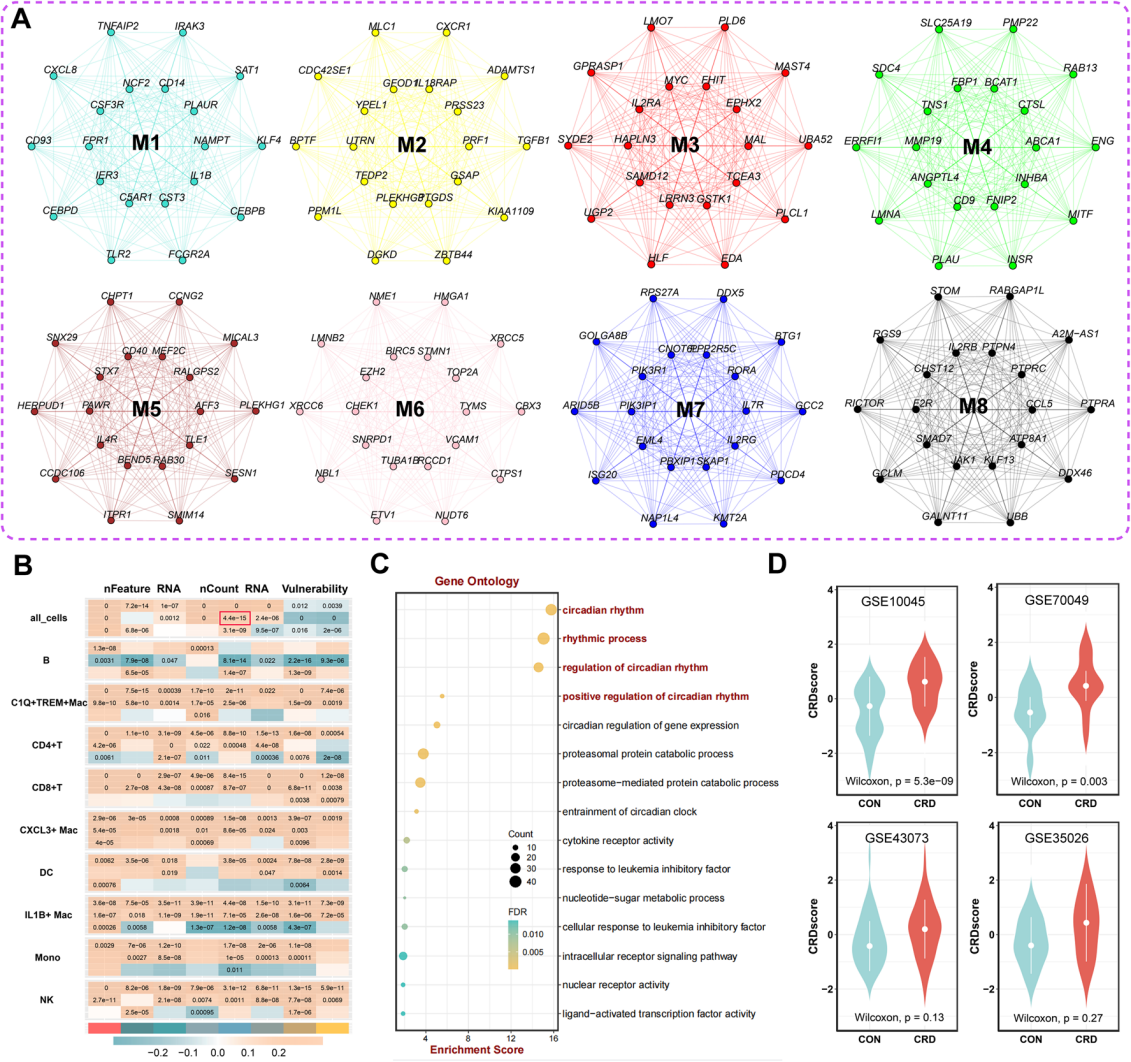
Identification of a circadian rhythm gene set associated with vulnerable plaques in coronary heart disease. (**A**) Visualization of hub genes of 8 modules in the co-expression network. (**B**) Correlation diagram of various immune cells with vulnerability. (**C**) Function enrichment analysis of M1 modules. (**D**) Box plots showing the relationship between two groups and CRDscore in the four GEO cohorts.

To identify the CRD genes associated with CAD, we first visualized the interaction network among hub genes in each module separately (Fig. 2A). The heatmap showed that all cells in the M1 module were the most relevant to vulnerability plaques (Fig. 2B). Gene Ontology (GO) enrichment analysis of hub genes from the M1 modules underscored their involvement in circadian rhythms (Fig. 2C).

CRDscore was calculated using the hub genes in the M1 module. Furthermore, we verified the performance and robustness of CRDscore using bulk transcriptomic datasets. In total, we obtained four independent gene expression datasets involving mouse models in the CRD group and control group. Employing the independent dataset, we identified different circadian rhythm patterns, with significantly higher CRDscore in the CRD group compared to the control group (Fig. 2D). These findings underscored the effectiveness of the CRDscore in accurately characterizing CRD status within bulk transcriptomic datasets.

### Identification of the CRD-related subtypes of CAD

**Figure 3:**
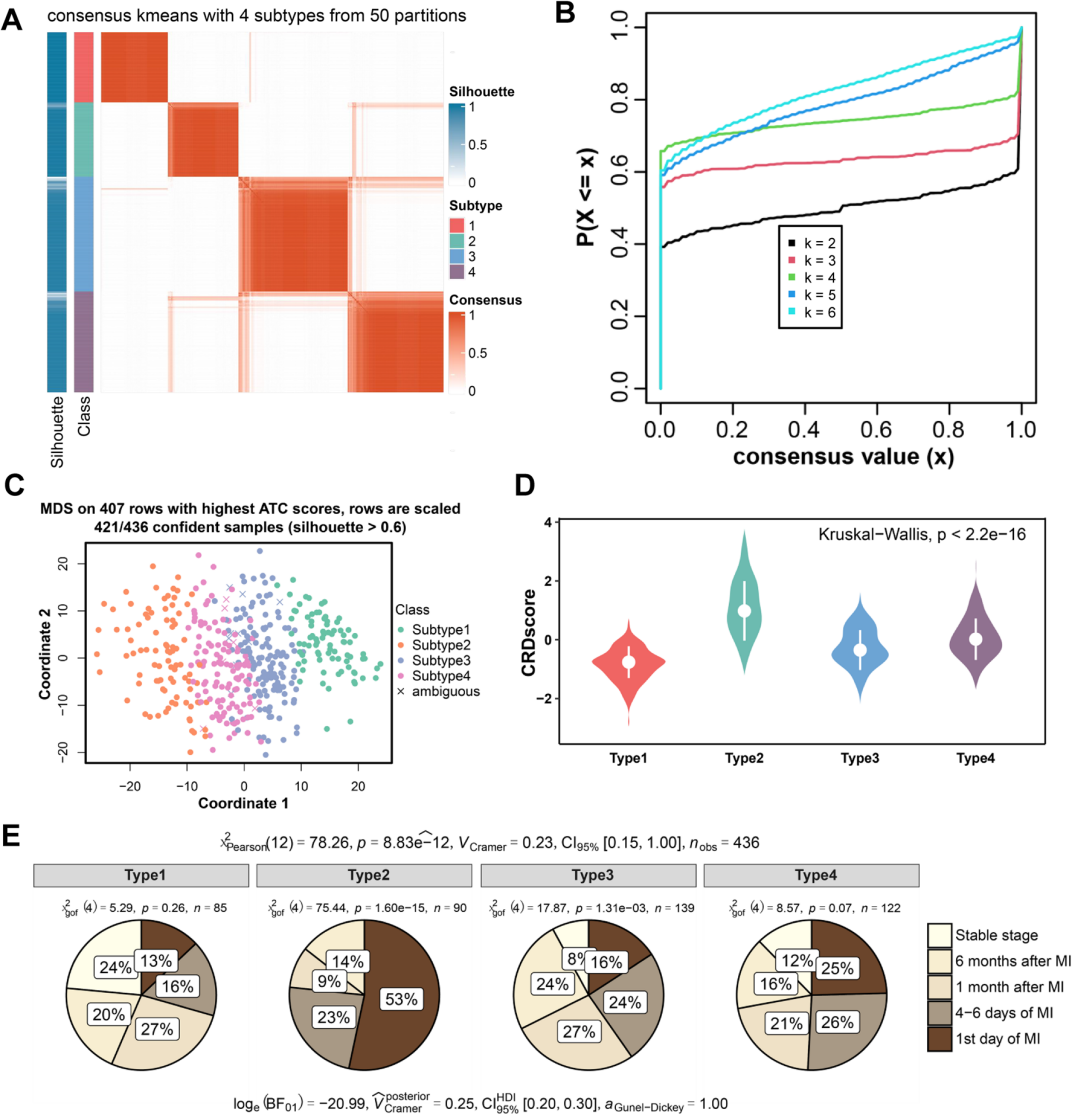
The construction of CRD molecular subtypes in the GSE59867 cohort. (**A**) Heatmap of consensus matrix when the total samples were divided into four subtypes. (**B**) CDF plot when the k value ranges from 2 to 6. (**C**) Clustering diagram for samples of four subtypes. (**D**) Box plots showing the relationship between four subtypes and CRD score. (**E**) Circle diagrams of the different stages of four subtypes.

We identified four robust subtypes in CAD based on consensus clustering using hub genes of M1 genesets in GSE59867 (Type1: 124 samples; Type2: 93 samples; Type3: 87; Type4: 132 samples). The boundary between heatmaps of the consistency matrix remained well-defined, demonstrating that the model categorization was rigorous and powerful (Fig. 3A). Moreover, we found that k = 4 was the optimal recommendation according to the consensus cumulative distribution function (CDF) curve (Fig. 3B). After the Coordinate dimensional reduction clustering of all samples, it was shown that the four subtypes of samples were divided into four different clusters with significant differences (Fig. 3C). We compared CRDscore for four subtypes, and Type2 had the highest CRDscore (Fig. 3D). It suggested that CRDscore was positively correlated with the severity of CAD. Besides, we divided patients into 5 groups according to their disease stage and described the relative proportion of 5 groups in each CAD subtype for the GSE59867 cohort (Fig. 3E). It could be seen that the group with severe disease stage accounted for the largest proportion in the Type2.

### The CRD-related subtypes validation in the GEO cohorts

**Figure 4:**
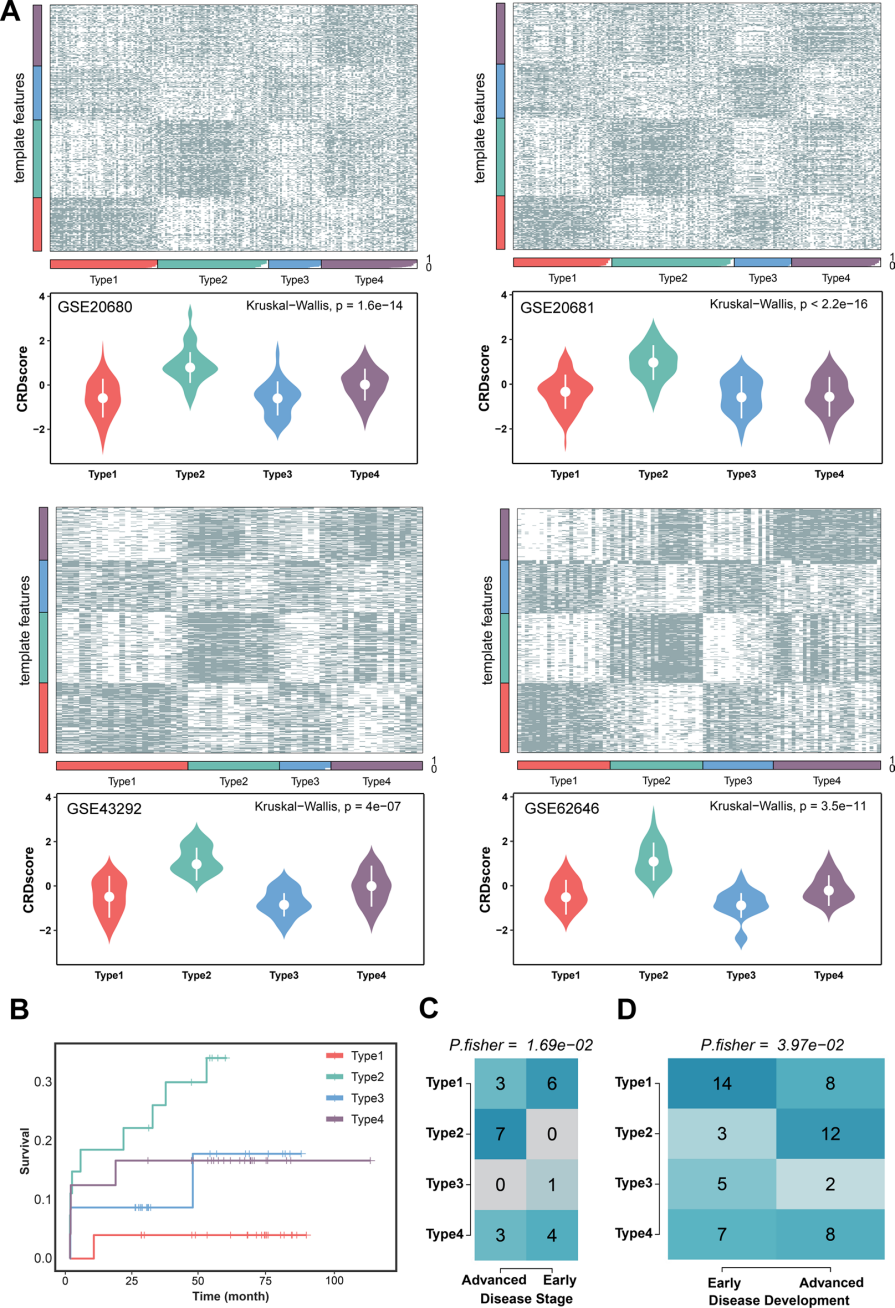
The validation of the stability of four subtypes by CRDscore. (**A**) The NTP heatmap and box plots with CED scores for four subtypes in the four GEO cohorts. (**B**) Survival analysis among four subtypes. (**C**) The heatmap of diverse disease stages among four subtypes. (**D**) The heatmap of diverse disease development among four subtypes.

The reliability and reproducibility of our taxonomy were validated using four GEO cohorts, namely GSE20680, GSE20681, GSE43292, and GSE62646. The key genes of four subtypes were incorporated into the NTP validation framework^29^. In these four cohorts, the four subtypes recognized in the GSE59867 cohort could be well reproduced and distinguished. Similarly, Type2 exhibited the highest CRDscore in testing cohorts (GSE20680 cohort: P = 1.6e-14; GSE20681 cohort: P < 2.2e-16, GSE43292 cohort: P = 4e-07, GSE62646 cohort: P = 3.5e-11, Fig. 4A), which aligned with our previous findings. Moreover, the heatmap also demonstrated the existence of four distinct subtypes in the four GEO cohorts. In the GSE59867 cohort, the comparison of survival also showed that patients in the Type2 group possessed dramatically worse outcomes than the other groups (Fig. 4B), which contradicted previous results. In addition, the heatmap showed the different disease stages and disease developments in the four subtypes (Fig. 4C,D). The Type2 subtype had the most severe disease stage and the most prominent deterioration of the disease.

### Biological peculiarities of each CRD-related subtype

**Figure 5:**
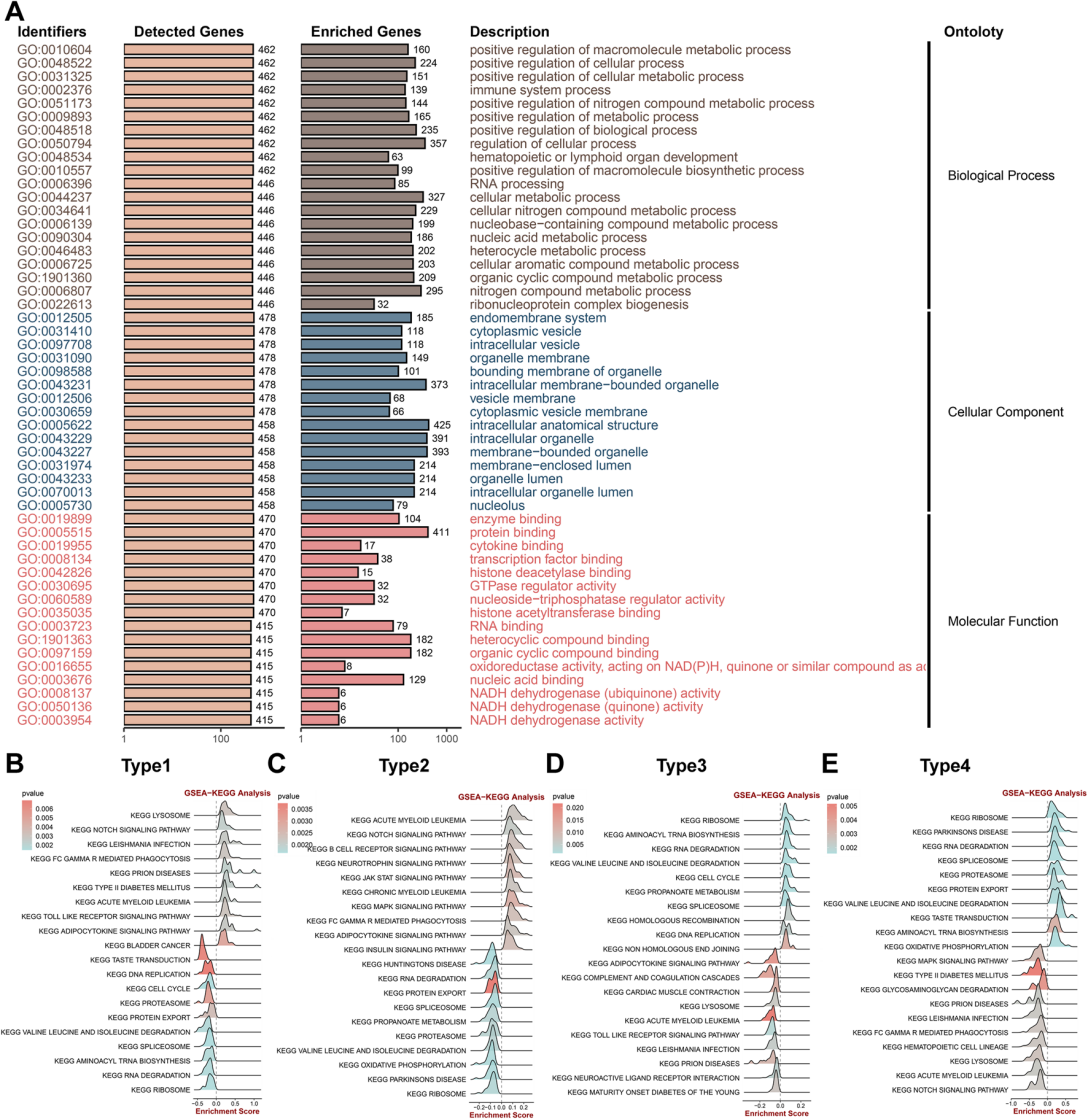
Biological functions of four subtypes. (**A**) Function enrichment analysis of Type2 subtypes. (**B**–**E**) Gene set enrichment analysis of four subtypes including Type1(**B**), Type2(**C**), Type3(**D**), Type4(**E**).

To further investigate the potential biological characteristics of distinct subtypes, a functional enrichment analysis was conducted (Fig. 5A–E). The GO enrichment analysis of Type2 exhibited significant enrichment in “positive regulation of macromolecule metabolic process”, “positive regulation of cellular process”, “positive regulation of cellular metabolic process”, “immune system process”, “endomembrane system”, “cytoplasmic vesicle”, “intracellular vesicle”, “organelle membrane”, “enzyme binding”, “protein binding”, “cytokine binding”, and “transcription factor binding” (Fig. 5A). Subsequently, gene set enrichment analysis (GSEA) was performed based on the markers of the four subtypes. Specifically, Type1 was distinguished by enhanced signaling pathway activation such as notch signaling pathway, Toll like receptor signaling pathway, and adipocytokine signaling pathway (Fig. 5B). Type2 demonstrated enhanced cell proliferation and immunoinflammatory pathways, including the regulation of B cell receptors, immune response mediated Fc-γ receptor, and JAK-STAT signaling pathway (Fig. 5C). It implied that Type2 might be primarily associated with the proliferation and immune activation of coronary atherosclerotic plaque cells. Type3 exhibited metabolic alterations in amino acid and RNA, along with the maintenance of cellular homeostasis. The pathways related to cellular homeostasis, such as amino acid synthesis, RNA breakdown and regulation, and DNA recombinational repair, were significantly upregulated in this subtype (Fig. 5D). On the other hand, Type4 was predominantly enriched in protein and RNA metabolism (Fig. 5E).

### Immune microenvironment profiles of four subtypes

**Figure 6:**
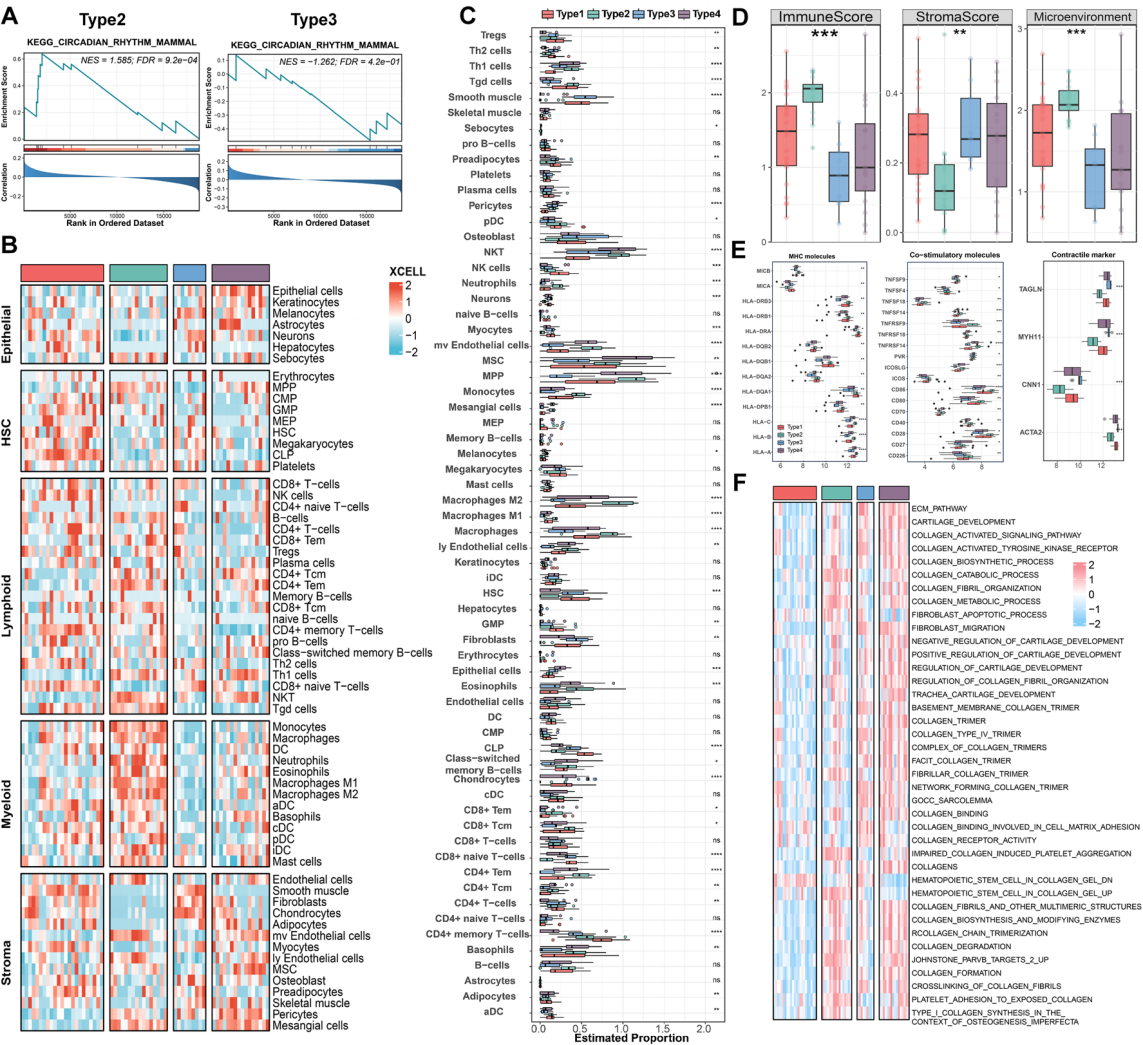
The distinct immune microenvironment patterns of four subtypes. (**A**) GSEA shows CRD-related gene sets enriched in Type2 and Type3. (**B**) Heatmap of expression levels of various cell types among four subtypes. (**C**) Box plot showing the estimated proportion of the various cell types among the four subtypes. (**D**) Immune score, stromal score, and microenvironment in four subtypes. (**E**) The expression level of MHC molecules, co-stimulatory molecules, and contractile markers in four subtypes. (**F**) Heatmap showing expression levels of four subtypes associated with fibrosis and collagen-related pathways.

According to preceding studies, immunoinflammatory pathways were found to be pronounced in Type2. Accumulating studies indicated that immune activation could lead to unstable changes in the lipid core of atherosclerotic plaque^19,39^. Therefore, we investigated the immune landscape of four subtypes to gain a better understanding of the etiopathogenesis of CAD. Figure 6A demonstrated that the circadian regulatory pathway was significantly enriched in Type2 and Type3, which was consistent with previous conclusions that CRD promoted poor prognosis in patients with CAD. Moreover, we conducted cell infiltration analysis and visualized the infiltration abundance of 64 cell types in each subtype using a heatmap (Fig. 6B,C). Compared with other subtypes, Type1 was mainly enriched in hematopoietic stem cells. As expected, Type2 displayed a higher level of leukocyte infiltration, including central memory CD4 + T cells, central memory CD8 + T cells, and various types of innate immune cells, particularly monocytes and macrophages. The abundance of immune cells suggested that they might have a better response to immunotherapy. Type3 and Type4 were all not significantly enriched in various cell types, especially in innate immune cells. It might be related to the better prognosis in Type3, although it was also upregulated in the circadian regulatory pathway. Besides, the immune score and microenvironment score of Type2 were significantly higher than those of other subtypes (Fig. 6D).

To further quantify the immune ecosystem across four subfamilies, an immune checkpoint analysis was executed (Fig. 6E). It was elaborated that Type2 presented the higher activation of both more MHC molecules (MICB, HLA-DRB3, HLA-DQB2, HLA-DPB1, HLA-C, HLA-B, etc.) and co-stimulatory receptors (e.g., TNFSF9, TNFSF18, TNFRSF9, TNFRSF14, ICOSLG, CD86, CD28), which might possess higher immune activation environment. However, contractile markers (TAGLN, MYH11, CNN1, ACTA2) were significantly down-regulated in Type2. Studies have shown that the degree of collagen fibrosis is positively correlated with the stability of CAD^40,41^. Therefore, we analyzed the differences in collagen synthesis and metabolism pathways in different subtypes (Fig. 6F). It showed that Type1 and Type2 had a low-level enrichment of collagen-related pathways, with the latter being associated with a poor prognosis. Therefore, further mechanistic studies of fibrosis will provide new insights into the precise treatment of CAD patients with low levels of collagen fibrosis.

## Discussion

Substantial evidence has shown that individuals who adhere to a healthy lifestyle experience a significant decline in the incidence of cardiovascular events^42^. The advocacy of wholesome lifestyle practices such as abstaining from smoking, avoiding obesity, and consistent physical exercise, is the heart of initiatives to bolster cardiovascular health among the population^43^. However, the intricate network of risk factors, varying treatment approaches, and diverse prognoses across different stages of CAD progression pose formidable obstacles to translating cutting-edge molecular biology discoveries into effective therapies for patients. Moreover, various experimental and clinical research has established that many cardiovascular parameters, such as heart rate, heart rate variability (HRV), electrocardiogram (ECG) waveforms, endothelial cell function, and blood pressure, exhibit robust circadian rhythms. These studies collectively demonstrated that CRD is associated with maladaptive cardiac functions, potentially precipitating adverse cardiovascular outcomes^16,44,45^. Therefore, the identification of specific biomolecular characteristics linked to the onset and progression of CAD is of utmost importance to both preventive approaches and the refinement of tailored therapies. In this study, we pinpointed a collection of circadian rhythm genes significantly correlated with the occurrence and progression of CAD, which we have referred to as the CADCR genes. Based on CADCR genes, CRDscore was calculated by computational biology algorithm. From both clinical and biological standpoints, evaluating the malignancy of CAD based on CRDscore may offer insights into prevention and the development of potential interventions.

Firstly, we identified the CADCRgenes associated with CAD using SCCNA in the scRNA-seq data and explored the CRDscore in the scRNA-seq data and bulk microarray data. In bulk microarray samples, the CRD group displayed a higher score, affirming the validity and consistency of the CRDscore as an assessment criterion. Next, four subtypes (Type1-Type4) based on the specific CRD-related genes were identified, and the four heterogeneous subtypes presented remarkable differences in the prognosis and clinicopathologic characteristics. The validation in bulk CAD samples revealed that this taxonomy possessed a superior extrapolation possibility. Subsequently, we delved into the relationship between CRDscore and other prognostic factors, encompassing disease stage and development. The results suggested that Type2 had the highest CRDscore and corresponded with the least favorable prognosis. Such insights emphasized the potential of the CRDscore as a predictive marker for the clinical management of CAD.

Understanding the distinct biological characteristics of these subtypes could guide personalized treatment strategies. Type1 was deemed as an immune-rejection/excluded subtype, with a low CRDscore, a favorable prognosis, abundant immune cells, high co-stimulatory, MHC molecule expressions, and low level of collagen metabolism. Type2 displayed malignant phenotypes with dismal clinical outcomes, with the worst prognosis. Type2 was significantly enriched in immunoinflammatory pathways, with a high CRDscore, high immune activation, and low level of collagen metabolism. Recent research has shown that inflammation plays a key role in coronary artery disease and atherosclerosis, in which immune mechanisms interact with metabolic risk factors to initiate, propagate, and activate lesions in the arterial branch^46,47^. Immune cells dominate early atherosclerotic lesions, their effector molecules accelerate the progression of the lesions, and activation of inflammation can elicit acute coronary syndromes^48–50^. Cardiac fibroblasts play a central role in maintaining homeostasis of the extracellular matrix (ECM). Fibroblast activation protein is expressed by myofibroblasts derived from smooth muscle cells in human atherosclerotic plaques and is involved in the pathogenesis and progression of atherosclerosis, particularly in inflammatory processes^51,52^. This promotes the deterioration and progression of CAD, which also matches that Type2 is predominantly in the advanced stage and development of CAD. In consideration of the high CRDscore and enriched immunoinflammatory pathways of Typ2, we hypothesized that circadian rhythms manipulation and immunosuppression treatment might be a novel latent therapeutic agent for these patients. Type3 and Type4 were characterized by metabolisms of amino acid and RNA, with a favorable prognosis, low CRD score, low immune cell infiltration, high contractile markers expressions, and enrichment of most collagen metabolism-related pathways. It can be assumed that Type3 and Type4 possessed a low immune activity. It also revealed the enrichment of collagen metabolism-related pathways in Type3 and Type4, which indicated a close correlation between the level of collagen fibrosis and the progression of this disease. For these patients, further research into the fibrotic mechanisms and the development of treatment methods to control fibrosis progression will benefit them.

Our study represents the pioneering effort to unveil CRD genes closely entwined with the process of CAD development at the single-cell level and bulk data. Employing diverse bioinformatics methodologies, we comprehensively delineated the expression landscape associated with circadian rhythms, ultimately establishing robust markers for predicting CAD development, guiding treatment decisions, and predicting prognosis. Taken together, we aspire to translate these findings into effective therapeutic modalities for the benefit of patients. Subsequent research endeavors will continue to be anchored in CRD genes, to develop minimally invasive early detection tools to curb the incidence of CAD.

## Conclusion

Collectively, we comprehensively and systematically explored the crosstalk between CRD and immunity in CAD, revealing their molecular characteristics and immune landscape in CAD. There was an interactive and intricate network of regulated fibrosis of tissue, immunity, and circadian rhythm disruption in CAD. Our platform preliminarily found that the higher CRDscore was closely related to higher immunoinflammatory activation and lower fibrosis, which could promote an unstable transformation of atherosclerotic plaque.

## Data Availability

Public data used in this work can be acquired from the Gene Expression Omnibus (GEO, http://www.ncbi.nlm.nih.gov/geo/).

## Abbreviations

CAD: Coronary atherosclerotic heart disease
CRD: Circadian rhythm disruption
CRDscore: Circadian rhythm disruption score
SCCNA: Single cell co-expression network analysis
MI: Myocardial infarction
scRNA-seq: Single-cell RNA sequencing
GEO: Gene expression omnibus
PCs: Principal components
UMAP: Uniform Manifold Approximation and Projection
t-SNE: T-distributed stochastic neighbor embedding
RMA: Robust multi-array averaging
TPM: Transcript per kilobase million
MEs: Module eigengenes
KME: The assessment of module eigengenes' interconnectivity
CADCRgenes: Coronary atherosclerotic heart disease circadian rhythm genes
CRG: Cell rhythmic gene
FDR: False discovery rate
SD: Standard deviation
PCA: Principal component analysis
DEG: Differential gene expression
GO: Gene Ontology
CDF: Cumulative distribution function
GSEA: Gene set enrichment analysis
BP: Blood pressure
HR: Heart rate
